# The use of ex-vivo normothermic perfusion for the resuscitation and assessment of human kidneys discarded because of inadequate in situ perfusion

**DOI:** 10.1186/s12967-015-0691-x

**Published:** 2015-10-16

**Authors:** Sarah A. Hosgood, A. D. Barlow, J. Dormer, M. L. Nicholson

**Affiliations:** Transplant Group, Department of Infection, Immunity and Inflammation, Leicester General Hospital, University of Leicester, Leicester, LE5 4PW UK; Department of Surgery, Addenbrooke’s Hospital, University of Cambridge, Cambridge, CB2 OQQ UK; Department of Medical and Social Care Education, University of Leicester, Leicester, LE1 9HN UK

## Abstract

**Background:**

Many kidneys are rejected for transplantation due to inadequate in situ perfusion during organ retrieval because of the risk of additional ischaemic injury and microvasculature thrombosis. This study describes the use of ex vivo normothermic perfusion (EVNP) for the resuscitation and assessment of human kidneys that were discarded after inadequate in situ perfusion.

**Methods:**

Twenty-two human kidneys were retrieved but then deemed unsuitable for transplantation, primarily due to inadequate in situ perfusion. After a period of static cold storage, kidneys were perfused for 60 min with an oxygenated red cell based solution at 36 °C.

**Results:**

Nineteen out of 22 kidneys (86 %) were from DCD donors. During EVNP, kidneys were assessed and scored based on their macroscopic appearance, measures of renal blood flow and urine production. Kidneys were scored from 1 indicating the least injury to 5, indicating the worst. Twelve kidneys had an EVNP score of 1–2, 7 scored 3–4 and 3 kidneys scored 5. The EVNP score 5 kidneys had a low level of tubular function compared to the score 1–4 kidneys. Their perfusion parameters did not improve during EVNP and they were considered non-transplantable. There was no association between the histological evaluation and EVNP parameters.

**Conclusion:**

EVNP restores function ex vivo and enables an assessment of kidneys that have been declined for transplantation due to inadequate in situ perfusion.

## Background

The successful utilisation of higher risk and donation after circulatory death (DCD) donor organs has undoubtedly contributed to the increase in the number of kidney transplants performed in the UK [1]. Nonetheless, there has been a steady increase in the number of kidneys donated but then subsequently discarded due to concerns about their quality [1]. Approximately 8 % of donation after brain death (DBD) and 18 % of DCD donor kidneys are retrieved but then declined for transplantation [1].

Inadequate in situ perfusion is a common cause of kidney discard, particularly in kidneys from DCD donors. It can be caused by donor factors such as atherosclerosis, difficult anatomy including multiple vessels or technical issues during retrieval. Inadequately perfused kidneys are likely to have additional warm ischaemic injury due to incomplete cooling during retrieval. The microcirculation is also likely to be compromised and the cumulative damage caused by a period of hypothermic preservation can result in irreversible injury [2]. Therefore, many of these kidneys are regarded as unsuitable for transplantation [3]. Ex-vivo normothermic perfusion (EVNP) can be used to recover function and assess the kidney prior to transplantation. An oxygenated red blood cell based solution is continuously re-circulated through the kidney at body temperature [4]. The perfusion conditions are designed to optimise recovery and promote repair mechanisms. The aim of this study was to use EVNP to resuscitate and evaluate kidneys declined primarily due to inadequate in situ perfusion and to assess their potential suitability for transplantation.

## Methods

### Ethics approval

From December 2012 to January 2014, 22 kidneys from the national organ allocation scheme were deemed unsuitable for transplantation primarily due to inadequate in situ perfusion and were recruited in this research project. Consent for the use of the organs for transplantation and research was obtained from the donor family by the Specialist Nurses in Organ Donation before organ retrieval. Ethical approval was granted for the study by the national research ethics commission in the UK.

### Kidney retrieval

Kidneys were retrieved by the UK National Organ Retrieval Service (NORS). The retrieval procedure for DCD kidneys was as follows. After asystole, a 5 min stand-off period was observed, followed by declaration of death. The patient was transferred to the operating theatre and a rapid laparotomy performed. The aorta was cannulated via the common iliac artery or aorta, and in situ cold perfusion commenced with University of Wisconsin (UW) or hyperosmolar citrate solution (HOC). The descending thoracic aorta was clamped and blood vented via the inferior vena cava. For DBD donors, laparotomy and warm mobilisation of abdominal organs was performed as appropriate. Following aortic cannulation, the supra-coeliac aorta was cross-clamped and cold perfusion commenced with venting via the inferior vena cava.

### Kidney donor information

The kidney donor information was recorded and included donor age, gender, ethnicity, cause of death, past medical history and terminal serum creatinine levels. The warm and cold ischaemic times, the type of preservation solution used during organ retrieval and the quality of perfusion was also recorded. The quality of in situ perfusion was assessed by the retrieval surgeon and documented as good = 1, fair = 2, poor = 3 or patchy = 4.

### Histological evaluation

A wedge biopsy was taken on arrival at the laboratory after the period of static cold storage. The tissue was fixed in 10 % formal saline then embedded in paraffin wax. Sections from the paraffin embedded tissue were cut (4 µm) and stained with Haematoxylin & Eosin (H&E) for histopathological scoring.

A consultant pathologist graded the sections using the Remuzzi score and assessed the level of acute tubular injury (ATI) [5]. Sections were graded mild, moderate and severe for the presence of ATI.

### Ex-vivo normothermic perfusion and functional analyses

Kidneys were prepared for EVNP and the renal artery, vein and ureter were cannulated.

The EVNP circuit was primed with a perfusate solution (Ringer’s solution, Baxter Healthcare) and supplements added to provide a physiological environment [4, 6, 7]. One unit of group 0 positive packed red blood cells from the local blood bank was added to the priming solution.

Kidneys were perfused with the red cell based solution at a set mean arterial pressure and near normal body temperature. Supplements were infused into the venous reservoir and arterial arm of the circuit to maintain normal homeostatic conditions as previously described [4, 6, 7].

Creatinine was added to the red cell based perfusate to allow an assessment of creatinine clearance and tubular function. The renal blood flow (RBF) and mean arterial pressure were recorded continuously and the mean calculated. Urine output was collected throughout perfusion and the total output measured. Blood gas analysis of arterial and venous blood was used to record acid–base homeostasis and measure oxygen consumption. Blood samples were taken pre-perfusion and after 60 min of EVNP. A urine sample was collected after 60 min of EVNP.

### EVNP assessment parameters

#### Macroscopic assessment

Each kidney was categorised into three groups according to its macroscopic appearance during EVNP as follows [8].

Grade I: Excellent perfusion (global pink appearance) (1 point).

Grade II: Moderate perfusion (patchy pink/purple appearance which either remained or improved during EVNP) (2 points).

Grade III: Poor perfusion (global mottling and purple/black appearance which remained throughout EVNP) (3 points).

### Functional assessment

The mean renal blood flow and total urine output during 60 min of EVNP was calculated and recorded.

### Derivation of EVNP assessment score

A combination of the macroscopic and functional parameters were used to create an index of organ quality. Receiver operating characteristic (ROC) curves were previously used to determine thresholds of renal blood flow and urine output to differentiate between macroscopic grades I and II versus grade III [8]. These thresholds were combined with the macroscopic grade to give an overall EVNP assessment score of 1–5. Macroscopic grades I, II and III were assigned points of 1, 2 and 3, respectively. Kidneys with a mean renal blood flow below the threshold (<50 ml) were given an additional score of 1. Kidneys producing less than the threshold of urine output (<43 ml) were also given an additional score of 1. Therefore, overall EVNP assessment scores ranged from 1 indicating the least injury to 5, the most severe (Table 1). This score provides a quantitative measure of functional parameters that support the macroscopic appearance.

**Table 1:** Ex-vivo normothermic perfusion (EVNP) assessment score

EVNP assessment	Point
Macroscopic assessment	
Grade I: Excellent perfusion (global pink appearance)	1
Grade II: Moderate perfusion (patchy appearance)	2
Grade III: Poor perfusion (global mottled and purple/black appearance)	3
Renal Blood flow	
Threshold ≥50 ml/min/100 g	0
Threshold <50 ml/min/100 g	1
Total urine output	
Threshold ≥43 ml	0
Threshold <43 ml	1

Macroscopic assessment, thresholds of renal blood flow and urine output. Scores ranges from 1 to 5, 1 indicating the least injury to 5 the most severe

### Statistics

Continuous data are presented as mean ± SD, and median (range) were appropriate. Data was compared using ANOVA with Tukey’s multiple comparison test or Kruskal–Wallis test with Dunns multiple comparisons test were appropriate. Categorical variables were analysed by Fisher’s exact test. P < 0.050 was considered statistically significant. Donor factors (age, retrieval creatinine, the warm ischaemic time, cold ischaemic time and perfusion grade at retrieval) were correlated with the EVNP score and the Remuzzi biopsy score using linear regression. GraphPad Prism 6 was used for statistical analysis (GraphPad Software, La Jolla, CA, USA).

## Results

All kidneys were declined primarily due to inadequate in situ perfusion. There were other contributing factors that added to the reason for decline in 9 of the kidneys; 3 due to the age of the donor, a pair due to a query on the histology report, a pair because the donor was anuric and 1 due to a history of drug abuse (Table 2).

**Table 2:** Donor and Kidney demographics

No	Donor	Gender	Donor type	Ethnicity	Cause of death	PMH	Retrieval	Kidney	Preservation WIT		CIT
	Age	M:F					Cr (µmol/l)	L:R	Solution	Min	(h)
Score 1 and 2											
2	66	F	DBD	White	ICH	HTN	61	L	HOC	0	25.30
3^a^	68	M	DCD	White	ICH	None	83	R	UW	10	28.02
5	65	F	DCD	White	Cardiac arrest	None	48	R	UW	15	34.11
6	55	M	DCD	White	ICH	Cardiac history, HTN	84	R	UW	11	20.28
7	71	F	DCD	White	ICH	CVA	58	R	UW	NR	32.12
11	54	M	DCD	White	Respiratory failure	CKD stage 2/3	108	L	UW	15	15.14
12	54	M	DCD	White	Respiratory failure	CKD stage 2/3	108	R	UW	15	16.24
14	76	M	DCD	White	ICH	Atrial fibrillation	76	R	HOC	12	13.00
15	41	M	DCD	Black	ICH	None	128	R	HOC	15	8.19
17	35	M	DCD	White	ICH	None	60	R	UW	12	33.54
18	35	M	DCD	White	ICH	None	60	R	UW	12	34.02
21^b^	40	M	DCD	White	Heart failure	Cardiomyopathy	58	L	UW	9	7.06
Score 3 and 4											
4	59	M	DCD	White	Respiratory failure	Pulmonary disease	102	R	UW	11	71.59
8^c^	46	M	DBD	Asian	Hypoxic brain injury	Cardiac arrest (60 min)	271	L	UW	0	27.52
9^c^	46	M	DBD	Asian	Hypoxic brain injury	Cardiac arrest (60 min)	271	R	UW	0	29.02
16	41	M	DCD	Black	ICH	None	128	L	HOC	15	9.39
19^a^	77	F	DCD	White	ICH	HTN	83	L	UW	12	26.30
20^a^	77	F	DCD	White	ICH	HTN	83	R	UW	12	27.39
22^b^	40	M	DCD	White	Heart failure	Cardiomyopathy	58	R	UW	9	8.15
Score 5											
1^d^	46	M	DCD	White	Pneumonia	Alcohol and drug abuse	153	R	UW	15	30.04
10	31	F	DCD	White	ICH	RTA	171	L	UW	9	19.34
13^a^	77	M	DCD	White	ICH	Headaches	49	L	UW	13	33.34

Donor demographics; age gender, donor type; donation after circulatory death (DCD) and donation after brain death (DBD). Cause of death; intracranial haemorrhage (ICH). Past medical history, retrieval creatinine, left or right kidney, preservation solution; University of Wisconsin (UW), hyperosmolar citrate (HOC), warm ischaemic time (WIT) and cold ischaemic time (CIT). Kidney numbers 8 & 9, 11 & 12, 15 & 16, 17 & 18, 19 & 20 and 21 & 22 were pairs from the same donor. CVA (cerebral vascular accident), CKD (chronic kidney disease), HTN (hypertension) RTA (road traffic accident), NR (not recorded)

Additional reasons for kidney decline ^a^Donor age, ^b^Histology, ^c^Donor anuric and ^d^History of drug abuse

The series included 6 pairs of kidneys. Three of the 22 kidneys (14 %) were from DBD donors and 19 (86 %) from DCD donors. Donor age ranged from 31 to 77 years. One pair of kidneys was included from an Asian donor and one pair from a Black donor. The ethnicity of the remaining donors was White. Three donors had a raised terminal serum creatinine (>132 µmol/l). The majority of the kidneys were flushed and stored in UW solution (19/22, 86 %). In the remaining kidneys, hyperosmolar (HOC) citrate was used. The warm ischaemic time (WIT) ranged from 9 to 15 min in the kidneys from DCD donors. The cold ischaemic time (CIT) ranged from 7 h 6 min to 71 h 59 min.

### Kidney evaluation and function

**Fig. 1:**
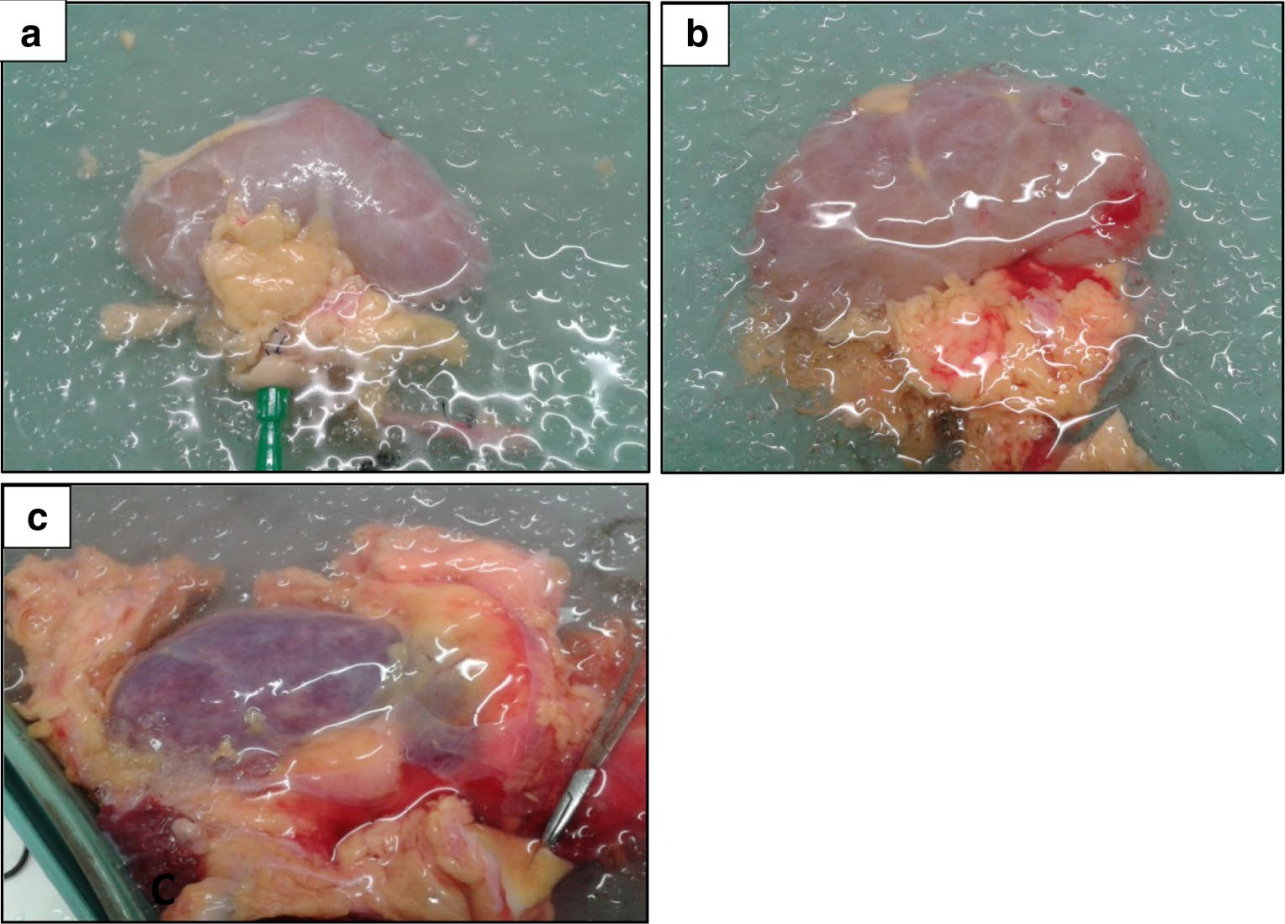
**a** Kidney with a mild patchy appearance after in situ cold perfusion and static cold storage. **b** Kidney with a moderate patchy appearance after in situ cold perfusion and static cold storage. **c** Kidney with a global purple appearance after in situ cold perfusion and static cold storage

The recorded quality of perfusion at retrieval ranged from good (1) to patchy (4). Using this system the retrieval surgeon graded kidney perfusion as good (n = 8), fair (n = 3), poor (n = 4) and patchy (n = 7) (Table 3). On arrival, and at the end of the static cold storage period, the macroscopic appearance of the kidneys and quality of perfusion ranged from those with a patchy appearance to those with a global purple appearance (Fig. 1a–c).

**Table 3:** Perfusion grade after retrieval (1–4), ex-vivo normothermic perfusion (EVNP) score, remuzzi score and level of acute tubular injury (ATI)

Kidney no	Perfusion grade	EVNP score	Remuzzi score	ATI
Score 1 and 2				
2	1	1	9	None
3	4	2	6	Moderate
5	4	2	4	Mild
6	2	2	3	Mild
7	1	1	7	None
11	1	2	10	None
12	4	1	6	Mild^a^
14	1	2	4	Mild to moderate
15	4	1	5	Mild to moderate^b^
17	1	2	–	–
18	1	2	0	Mild
21	3	2	3	Mild
Score 3 and 4				
4	4	4	2	None
8	2	3	3	Mild
9	2	3	1	Mild
16	4	3	6	None
19	1	3	2	Mild
20	4	3	5	Mild
22	3	3	–	Moderate
Score 5				
1	1	5	0	None^a^
10	3	5	–	Severe
13	3	5	6	None^c^

A wedge biopsy was taken on arrival at the laboratory after the period of static cold storage. The tissue was fixed in 10 % formal saline then embedded in paraffin wax. Sections from the paraffin embedded tissue were cut (4 µm) and stained with H&E for histopathological scoring. Sections were assessed using the Remuzzi score by a consultant pathologist who was blinded to the donor types. Four different parameters were assessed in the scoring system; Glomerular global sclerosis, tubular atrophy, interstitial fibrosis and vascular lesions (8). The score ranged from a minimum of 0 (indicating the absence of renal lesions) to 3 (severe). The sum of the four parameters was then calculated. A score of 0–3 indicated mild changes, 4–6 moderate and 7–12 severe. Sections were graded mild, moderate and severe for the presence of acute tubular injury. Pairs of kidneys; (8, 9), (11, 12), (15, 16), (17, 18), (19, 20) and (21, 22)

^a^Vacuolation of the proximal tubular epithelial cells

^b^Glomerular capillary thrombi

^c^Glomerular capillary thrombi and early cortical necrosis

**Fig. 2:**
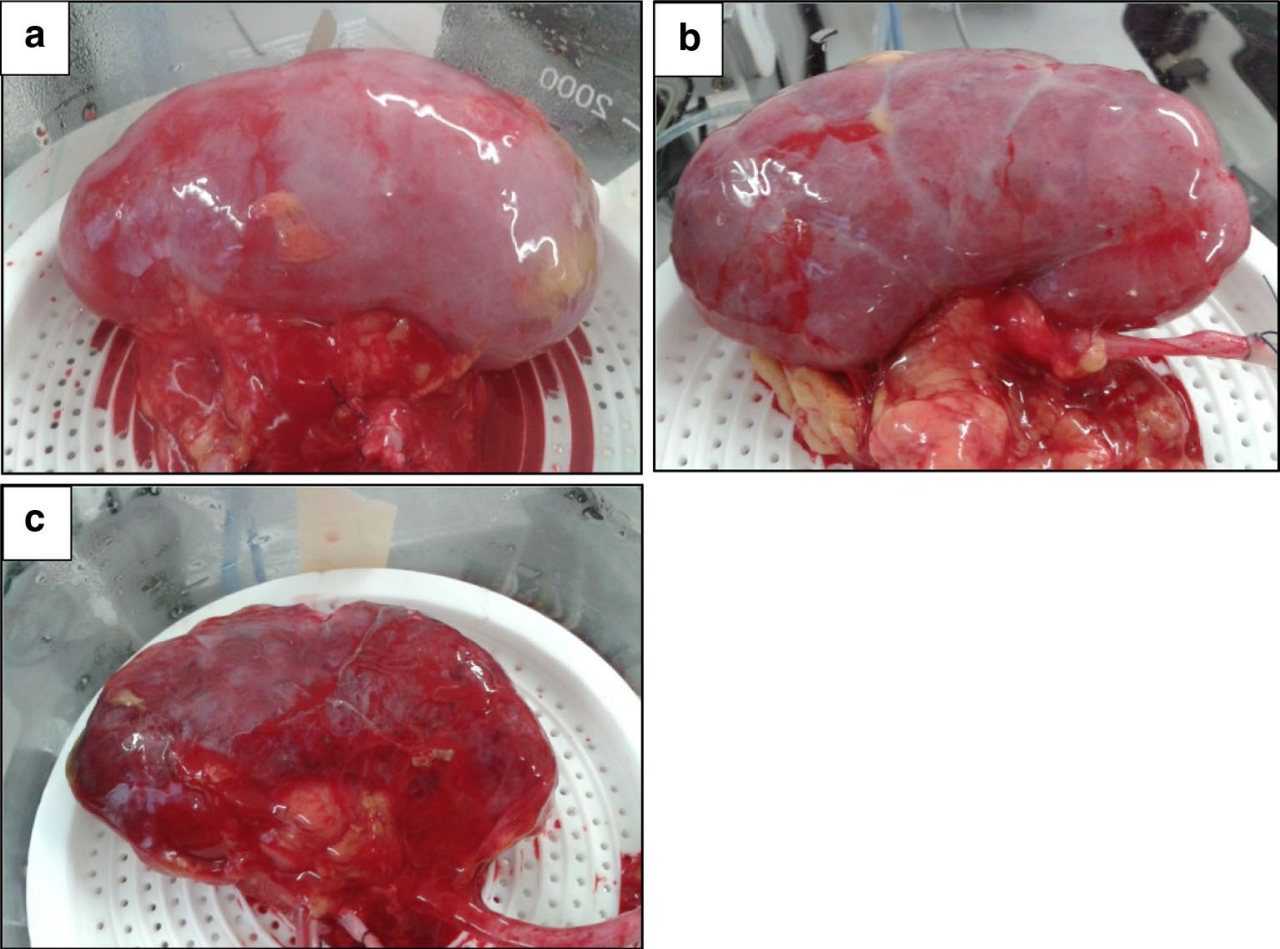
**a** Kidney with a global *pink* appearance* 1* during ex vivo normothermic perfusion. **b** Kidney with a patchy appearance * 2* during ex vivo normothermic perfusion. **c** Kidney with a global *purple/black* appearance * 3* during ex vivo normothermic perfusion

Application of the EVNP scoring system yielded the following scores: score 1 (n = 4), score 2 (n = 8), score 3 (n = 6), score 4 (n = 1) and score 5 (n = 3) (Fig. 2a–c). Kidneys were grouped into score 1 or 2 (n = 12), score 3 or 4 (n = 7) and score 5 (n = 3) (Table 3).

**Fig. 3:**
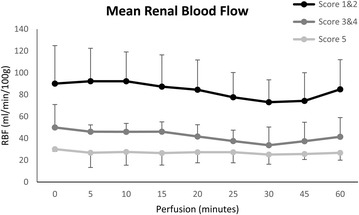
Mean renal blood flow during 60 min of ex vivo normothermic perfusion (EVNP). Kidneys were divided into three groups, EVNP score 1–2, EVNP score 3–4 and EVNP score 5. The mean renal blood flow was significantly higher in the score 1–2 vs 3–4 vs 5 kidneys and in the score 3–4 vs 5 (P < 0.0001)

The mean renal blood flow remained steady in the majority of kidneys during EVNP. The EVNP score 1–2 kidneys had a higher level of renal blood flow compared to the score 3–4 and score 5 (P < 0.0001; Fig. 3). The EVNP score 3–4 kidneys had a higher level than the score 5 (P < 0.0001; Fig. 3).

There were no significant differences in donor ages (mean ± SD) in the three groups of kidneys: score 1–2, 55 ± 15 years, score 3–4, 55 ± 16 years and score 5, 51 ± 24 years (P = 0.934). The mean CIT was 22.3 ± 10.2 h in the score 1–2 kidneys, 21.3 ± 8.9 h in the score 3–4 kidneys and 27.6 ± 7.3 h in the score 5 kidneys (P = 0.629).

**Fig. 4:**
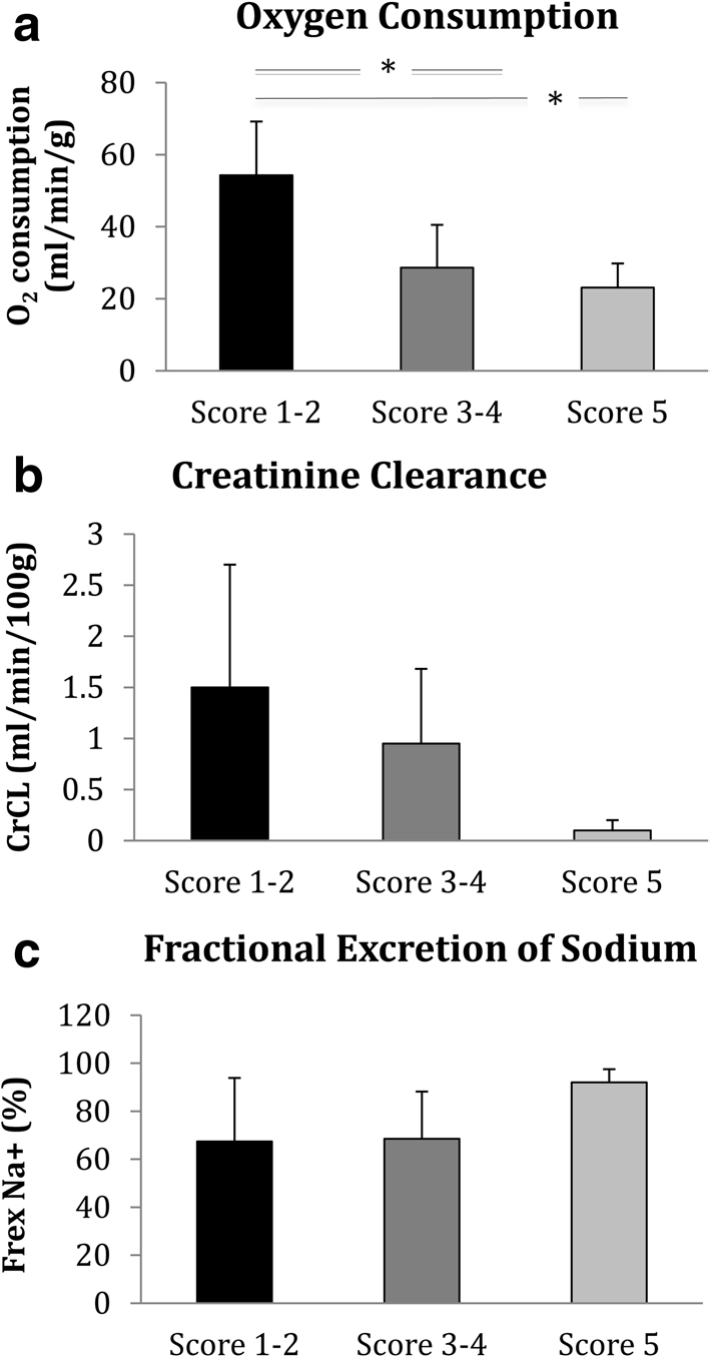
**a** Levels of oxygen consumption after 60 min of ex vivo normothermic perfusion (EVNP) in kidneys with an EVNP score of 1–2, 3–4 and 5. (*P = 0.006). **b** Levels of creatinine clearance after 60 min of ex vivo normothermic perfusion (EVNP) in kidneys with an EVNP score of 1–2, 3–4 and 5. **c** Levels of fractional excretion of sodium after 60 min of ex vivo normothermic perfusion (EVNP) in kidneys with an EVNP score of 1–2, 3–4 and 5

The level of oxygen consumption was significantly higher in score 1–2 kidneys compared to the score 3–4 and score 5 kidneys (P = 0.0006, respectively; Fig. 4a). The score 5 kidneys had a numerically lower level of creatinine clearance than the score 1–2 and score 3–4 kidneys although this did not reach statistical significance (P = 0.087; Fig. 4b). Tubular injury was highest in the score 5 kidneys, but again this did not reach statistical significance (P = 0.298, Fig. 4c).

There was no significant correlation between the EVNP score and donor age (P = 0.610), retrieval creatinine (P = 0.098), perfusion grade at retrieval (P = 0.732), the warm ischaemic time (P = 0.272) and the cold ischaemic time (P = 0.081).

### Histology

Three kidneys were not assessed due to an absence of glomeruli in the sample. There was no significant difference in the level of injury assessed by the Remuzzi score between the groups of kidneys (P = 0.476; Table 3). The Remuzzi scores ranged from (0 to 10) in the score 1 and 2 kidneys, (1–6) in the score 3 and 4 kidneys and (0 and 6) in the score 5 kidneys.

**Fig. 5:**
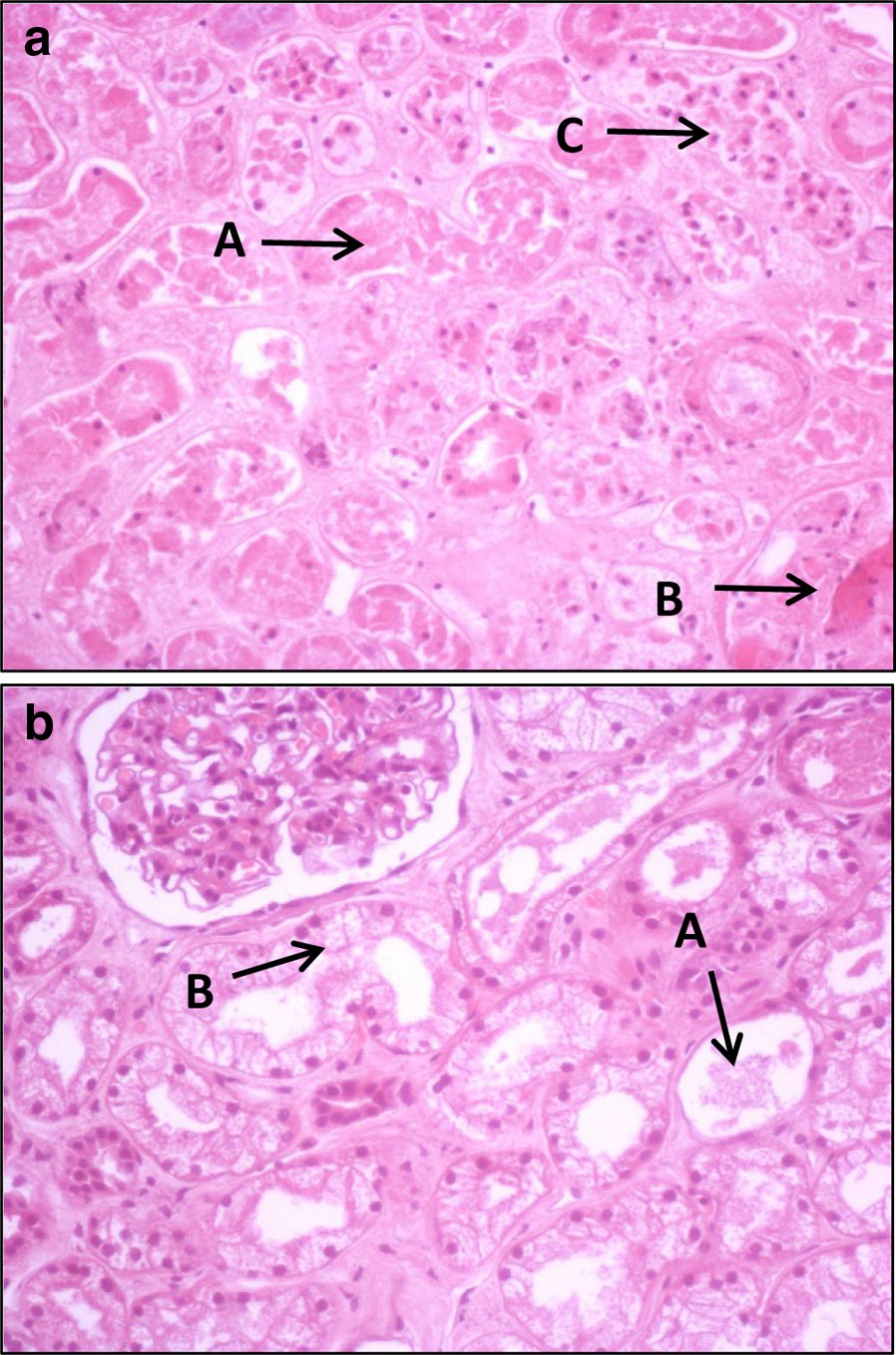
**a** Kidney number 10. Histology showing severe tubular injury (*A*), glomerular thrombi (*B*) and early cortical necrosis (*C*). **b** Kidney number 43. Histology showing moderate tubular injury (*A*) and vacuolation (*B*)

There was also no significant difference in the incidence and severity of acute tubular injury in the biopsy samples. In the majority of kidneys, injury ranged from zero to moderate ATI. Only one of the score 5 kidneys had severe ATI, with evidence of early cortical necrosis accompanied by glomerular capillary thrombi (Fig. 5a; Table 3). One kidney had glomerular capillary thrombi and 2 had significant vacuolation of the proximal tubular epithelial cells (Fig. 5b; Table 3).

There was no significant correlation between the Remuzzi score and donor age (P = 0.151), retrieval creatinine (P = 0.212), perfusion grade at retrieval (P = 0.709), the warm ischaemic time (P = 0.265) and the cold ischaemic time (P = 0.190). There was also no significant correlation between the EVNP score and Remuzzi score (P = 0.05).

Four of the six pairs had similar EVNP scores. The remaining two pairs scored 2 and 3 and 1 and 3 (Table 3). There were also differences in the Remuzzi scores in these two pairs and in a third (Table 3).

## Discussion

Function may be recovered in kidneys declined for transplantation due to inadequate in situ perfusion using EVNP. Cooling an organ to reduce cellular metabolism is an essential part of kidney preservation. Metabolism is slowed by a factor of 1.5–2 for every 10 °C drop in temperature [9, 10]. Flushing the organ with preservation solution at 4 °C ensures rapid cooling and clearance of the microcirculation. A well flushed kidney will have a uniform pale appearance, whereas those that are not adequately perfused may have a patchy or a global purple appearance. Inadequate in situ perfusion affects a large proportion of DCD kidneys and this was reflected in this series with the majority of these discarded kidneys being from DCD donors. Poor perfusion can be the result of donor factors such as arteriosclerotic occlusive disease or renal artery stenosis, improper placement of the aortic cannula, renal vasospasm or intravascular thrombosis. It results in a more prolonged warm ischaemic interval and the consequence of this in combination with a period of hypothermic preservation is a high level of cellular injury. Gok et al. reported in a small series of kidneys an exceptionally high rate of primary non function from inadequately perfused kidneys (66.7 %) compared to 4.8 % in those with adequate perfusion [2]. Furthermore, Snoeijs et al. found that the prognosis of kidneys that were not rapidly perfused due to prolonged catheter insertion or technical difficulties was associated with a poorer prognosis [11].

In this present series the quality of inadequate perfusion during organ retrieval varied significantly ranging from kidneys with small areas of patchy perfusion to those with a global purple appearance. The donor characteristics were also varied and in several of the kidneys other factors contributed to the decision not to transplant the organs. To address the high rate of kidney discard, Callaghan et al. recently assessed 20 kidneys that had been retrieved for transplantation but then deemed unsuitable by centres in the UK [3]. The evaluation was based on the macroscopic appearance and gross anatomy of the kidneys after a period of static cold storage. Three independent consultant transplant surgeons assessed each kidney. ‘Poor perfusion’ was the commonest reason for decline (25 %). The authors made no comment specifically on the severity of inadequate in situ perfusion. However, they did judge 4 kidneys to be useable and 9 possibly useable after histological assessment. A macroscopic assessment of the kidney before and after retrieval is an important step to rule out any gross abnormalities, evidence of inadequate perfusion or technical damage. In this present series 8 kidneys were judged as having good perfusion but were subsequently discarded on based on poor perfusion. This highlights the significant variability in judgement when assessing the quality of these kidneys. Furthermore there was no association between the grade of perfusion at retrieval and the EVNP score.

EVNP has the benefit of restoring function ex vivo, which has several advantages. The EVNP conditions are designed to be protective without the risk of exposure to leucocytes, complement or inflammatory mediators. Kidneys are perfused for 60 min with an oxygenated red cell based solution after hypothermic preservation. This primes and reconditions the kidney, reversing some of the detrimental effects of ischaemic injury [12]. Despite the fact that cold ischaemic times were prolonged in many of the kidneys in this series, the majority regained a good level of function. We applied a novel scoring system based on a combination of the macroscopic appearance of the kidney during EVNP and thresholds of renal blood flow and urine output (8). Overall 19 kidneys had an EVNP assessment score of 1–4 and 3 had a score of 5. A score of 1 indicated the least injury and a score of 5 the most severe. The score 5 kidneys appeared globally purple/black in appearance had an extremely low level of renal function, oxygen consumption and renal blood flow. Furthermore, they had an extremely low level of tubular function with no improvement during EVNP. We considered these score 5 kidneys unsuitable for transplantation. We have recently published the outcome of EVNP score 1–3 kidneys from our clinical series. Four out of 36 kidneys had delayed graft function (DGF) and there were no incidences of primary non function (PNF) [8]. We believe that EVNP score 4 kidneys may also be suitable for transplantation and therefore in this discarded kidney series 19/22 (82 %) were potentially useable. Of note several kidneys had a prolonged cold ischaemic time, which is likely to have a significant impact on graft function.

The series included 6 pairs of kidneys. Four of these pairs were identical but the remaining two pairs had different EVNP scores. Generally, studies of paired kidneys from deceased donors show similar outcomes [13]. However, there can be variations between kidneys due ischaemic injury or conditions during retrieval. The histological evaluation also showed some differences between the pairs. Kasiske et al., found significant variations between first and second biopsies from the same kidney and from the contralateral kidney in study of 83 pairs of kidneys, one of which was discarded and the other transplanted [14]. The discrepancies may have been due to the quality of biopsy or the experience of the observer [14–16]. Wedge biopsies were used in this present study but core biopsies may give a more accurate representation of the kidney [14] which may account for the differences.

The histological evaluation showed that only one kidney, with an EVNP score 5 kidney, had evidence of cortical necrosis. The donor had been involved in a road traffic accident with severe abdominal trauma. An extensive retroperitoneal haematoma was found at retrieval. Based on the Remuzzi score, only 3 kidneys would have been discarded, 8 were suitable for a single and 8 suitable for a dual transplant [5]. Histological evaluation using the Remuzzi score and assessing the level of acute tubular injury is useful in determining the level of damage and can be predictive of graft outcome [8, 14–16]. However, it is not necessarily associated with graft function [16]. Reliance on the histological evaluation to determine the suitability of a kidney for transplantation can lead to a high rate of unnecessary discard [14, 17]. Although the EVNP score was not associated with the histology evaluation, EVNP is likely to be a valuable asset in the decision process. This study analysed a small number of kidneys. A large multi-centre trial is planned to assess EVNP in DCD kidneys. A histological analysis will be included in the study and this we help us to understand the relationship between parameters during EVNP, histological evaluation and graft outcome.

The study was limited to perfusing and assessing these kidneys in the laboratory setting but expands on our previously published case report of a pair of kidneys recovered after in situ perfusion [18]. Without transplanting these kidneys it not possible to fully determine their outcome. We believe a short perfusion period is adequate to assess and prime a kidney. However more prolonged perfusion periods may be necessary for kidneys with more severe damage in order to allow recovery before transplantation.

## Conclusion

EVNP facilitates the recovery and assessment of kidneys that have been declined for transplantation due to inadequate in situ perfusion. This series suggests that a high proportion of these kidneys may be suitable for transplantation.
